# Development of Machine‐Assisted, Human‐Centred Bone Marrow Cell Classification: Feasibility Analysis in Patients With Myelodysplastic Syndromes

**DOI:** 10.1002/jha2.70205

**Published:** 2025-12-16

**Authors:** Kiyoyuki Ogata, Yuto Mochimaru, Leonie Saft, Gerrit Sijm, Bouchra Badaoui, Canan Alhan, Carole Almire, Yosuke Kato, Daisuke Sakamoto, Naoko Chiba, Naho Murata, Nana Kasai, Kazuma Sei, Naoya Kawahara, Mika Ogata, Nicolas Chapuis, Orianne Wagner‐Ballon, Arjan van de Loosdrecht, Yumi Yamamoto

**Affiliations:** ^1^ Department of Haematology Metropolitan Research and Treatment Centre For Blood Disorders (MRTC Japan) Tokyo Japan; ^2^ Clinical Pathology and Cancer Diagnostics Karolinska University Hospital and Institute Stockholm Sweden; ^3^ Department of Haematology Amsterdam UMC, VU University Medical Center Cancer Center Amsterdam Amsterdam the Netherlands; ^4^ Department of Haematology and Immunology Henri‐Mondor Hospital AP‐HP Créteil France; ^5^ Assistance Publique‐Hôpitaux de Paris. Centre‐Université Paris Cité Service d'hématologie biologique, Hôpital Cochin Paris France; ^6^ Department of Medical Technology Kyorin University Faculty of Health Sciences Mitaka Tokyo Japan; ^7^ Department of Clinical Laboratory Kyorin University Hospital Mitaka Tokyo Japan

**Keywords:** bone marrow, cytomorphology, digital images, myelodysplastic neoplasms, myelodysplastic syndromes

## Abstract

**Background:**

Cytomorphological examination and classification of bone marrow (BM) cells using a microscope is essential for diagnosing various diseases affecting the haematopoietic system. However, this requires expertise, is effort‐intensive and is inherently subjective.

**Methods:**

We developed a method for automatically capturing BM cell images at the single‐cell level using an existing cell image analyser designed for peripheral blood. The captured BM cell images were pre‐classified by the same analyser and subsequently viewed on a large screen by cytomorphologists for BM cell classification. The data consistency in BM blast percentages between the digital and conventional optical microscopy (CM) methods was examined.

**Results:**

During the 3.5‐year study period, 657 (96.2%) of the 683 aspirated samples underwent BM cell classification using the digital method. Digital cell images were captured at 2000× magnification and stored permanently, which allowed high‐quality cell image analysis at any time by anyone. Cell images could be compared side‐by‐side across different cell classes, which improves the cell‐classification accuracy. The detailed cell morphology in some cases differed slightly between the digital images and CM. BM blast percentage was comparable between the developed method and CM when examining patients with myelodysplastic syndromes (MDS) and other conditions that need to be differentiated from MDS. In a multicentre study, cytomorphologists who used this method for the first time could perform cell classification.

**Conclusion:**

This machine‐assisted and human‐centred approach enhances objectivity in BM cell classification, aligns with the needs of the digital era and facilitates data sharing.

**Trial Registration**: The authors have confirmed clinical trial registration is not needed for this submission.

## 1 Introduction

Cytomorphological examination of bone marrow (BM) cells using a microscope is essential for diagnosing and monitoring various malignant and non‐malignant diseases affecting the haematopoietic system. Notable examples include diagnostic and post‐chemotherapeutic follow‐up tests for myelodysplastic syndrome (MDS, also known as myelodysplastic neoplasm) and acute leukaemia [1, 2, 3]. In certain diseases, other diagnostic methods play a vital role, such as genetic analysis in chronic myeloid leukaemia and acute promyelocytic leukaemia. However, BM cytomorphology remains a crucial diagnostic tool, even in such cases [4]. The cytomorphological examination/classification of BM cells requires expertise, is labour‐intensive and is inherently subjective [5, 6, 7, 8, 9]. Mastering this skill is complex and demands extensive training under experienced professionals and is becoming less common in certain regions [10]. For instance, the BM aspiration procedure itself and examination of BM smears are not performed by many young haematologists in the United States [11], and poor‐quality BM smears (for example, massively haemodiluted smears) are prevalent and often overlooked by many haematologists in Japan [12].

Recently, artificial intelligence (AI)‐based automatic BM cell classification has shown promising results [13]. The major setbacks of AI‐based methods include the fact that human experts must confirm the results created by AI, and there is a substantial lag between the emergence of new disease entities and the AI's ability to adapt. In addition, the current AI‐based models struggle to accurately classify myeloblasts (Mbls), particularly in patients with MDS [14, 15], where Mbl morphology often deviates from typical patterns. These findings emphasise the importance of cytomorphological expertise among diagnosticians.

An alternative approach involves capturing snapshots of the BM smears on a personal computer (PC) and classifying the observed BM cells on the PC screen [16, 17]. However, this approach has not been routinely applied. One of the authors' institutions (MRTC Japan) developed a technique that can automatically capture BM cell images at the single‐cell level using an existing automatic cell image analyser for peripheral blood (PB). The captured BM cell images, which were pre‐classified by the same analyser, can be used to easily complete BM cell differential count by humans on a large PC screen. MRTC Japan is using this digital method as a supplementary differential count methodology to complement the standard optical microscope examination. The latter remains mandatory for analysing BM details other than the differential count, such as dysplasia and the presence of focal disease.

This study is the first to present our method and assess data consistency in BM cell differential count, particularly blasts (one of the key diagnostic metrics in MDS), between two approaches (our digital‐based method and conventional optical microscopy ‘conventional method’).

## 2 Materials and Methods

### 2.1 Subjects

We analysed all BM aspirations performed between January 2021 and June 2024 at MRTC, Japan, for clinical purposes. As MRTC Japan is a referral centre for patients with MDS, mainly those in advanced disease stages, most of the present BM samples were from patients with MDS who frequently underwent multiple BM examinations to assess disease status and response to chemotherapy [12, 18]. The diagnoses were made according to the 2016 World Health Organization criteria [19]. MDS cases with a megakaryoblastic immunophenotype were diagnosed based on our previous reports [18]. In certain cases, the presence of haematogones was confirmed using flow cytometry [20]. This study was approved by the Institutional Review Board of MRTC Japan (IRB189), and the procedures were performed in accordance with the Helsinki Declaration. Written informed consent was obtained from all the patients.

### 2.2 BM Cell Sample Processing and Capturing Cell Images Using CellaVision

This process is summarised in Figure S1. A small volume (0.3–0.5 mL) of BM cells was aspirated from the iliac crest using a standard method with a disposable syringe, without anticoagulant. Immediately, half the volume of the aspirated sample was used for smear preparation via the wedge method (referred to as the standard BM smear in this study). In contrast, the remaining half was transferred into a mini tube (CJ‐2DK, TERUMO, Tokyo, Japan), which is designed for collecting a small volume (0.25–0.5 mL) of PB and contains dipotassium ethylenediaminetetraacetic acid (EDTA‐2K), and mixed thoroughly using a disposable dropper (bore diameter 1.8–2.0 mm; Asiakizai, Tokyo, Japan). Standard BM smears were stained using the Wright–Giemsa solution. Simultaneously, using the EDTA‐2K anticoagulated BM cells, we manually prepared four smears from each sample using the wedge method (BM smears for CellaVision analysis [called CV BM smears]) as follows: a portion of the anticoagulated BM cells was typically diluted two‐fold (occasionally up to 3‐fold when the samples were extremely hypercellular) with plasma from the same patient. Because the CellaVision system requires erythrocyte monolayers to capture images of nucleated cells, excessive dilution was avoided to ensure adequate monolayer formation. Plasma was obtained from EDTA‐2K anticoagulated PB samples, collected on the same day as BM aspiration for routine clinical tests. Two smears were prepared from diluted BM samples, while the remaining two smears were prepared from undiluted BM samples. We made these CV BM smears more widespread than standard BM smears for efficient cell image capture. All CV BM smears were stained with Wright–Giemsa solution using an ADVIA autoslide stainer (SIEMENS, Munich, Germany) within 1 h of BM aspiration and analysed using CellaVision DM1200 (CellaVision, Lund, Sweden). The types of cell classes prepared as various cell folders in the original CellaVision DM1200 program were insufficient for comprehensive BM cell classification. Therefore, prior to initiating this study, we created new cell folders to match BM cells, including various stages of erythroblasts (Ebls), immature monocytes and promonocytes.

### 2.3 BM Cell Classification Using Digital Image

The CellaVision DM1200 automatically captured cell images from smears at 2000× magnification (the machine was designed to capture one nucleated cell per image in ideal conditions) and simultaneously classified the cell images based on classification algorithms for PB and created a database for each smear. Four databases were generated for each case. We reviewed all four databases and selected the most suitable one for final classification based on the following criteria: 1) Most images contained a single nucleated cell. 2) Sufficient number of nucleated‐cell images (approximately 500 nucleated cells) were captured. The rationale for the first criterion is described in Text S1.

In the automatic classification using CellaVision, each cell image was stored in a single‐cell folder. However, classification errors were observed, including a proportion of cells labelled as ‘Unclassifiable,’ smudge cells appearing in multiple cell folders, and absence of cells in newly created cell folders. On a 27‐inch PC monitor (display resolution, 3820 × 2160 UHD) that was connected to the CellaVision DM1200, manual revisions were performed according to the manufacturer's instructions. For instance, cell images can be moved by reassigning them to appropriate folders via drag‐and‐drop. In MRTC Japan, this revision was performed first by an experienced cytomorphologist (YM) and then confirmed and revised again, if necessary, by another experienced expert (KO) with YM (see Text S2 for more details).

### 2.4 Concordance Between Digital and Conventional BM Cell Classification Methods

All BM samples during the study period were examined using the aforementioned method at MRTC Japan. We assessed data consistency between the digital and conventional classification methods for the same BM samples examined by the same evaluators.

First, cases in which BM cell classification could not be performed using the digital method (mainly because of dry taps) were excluded. For the concordance analysis at MRTC Japan, 100 consecutive standard BM smears from patients with MDS, including leukaemic cases, obtained from January 2023 to June 2023, were anonymised and classified using the conventional method (500 cells/sample). Similarly, 37 standard BM smears, obtained from 28 patients with non‐clonal diseases and six patients with myeloproliferative neoplasms (MPNs) during the study period (one patient with an accelerated phase of chronic myeloid leukaemia was excluded), were anonymised and subjected to cell classification using the conventional method. This classification was performed by two individuals (Y.M. and K.O.) who had previously performed BM cell classification using a digital method.

We then designed a multicentre study, inviting cytomorphologists from an institution in Tokyo and all members of the European LeukemiaNet iMDS flow cytometry working group, and seven of them participated. In this multicentre study, patients with MDS with varying percentages of Mbl in the BM who were examined at MRTC Japan were assigned to each participating cytomorphologist (six patients for the initial two participating cytomorphologists and five patients for the other participating cytomorphologists). The overlap of patients among participating cytomorphologists was minimal. Digital images of these patients, which were automatically classified using CellaVision, were stored on a PC at MRTC Japan, where the CellaVision DM software was installed. Using a remote access software (Splashtop Personal, Splashtop Inc., CA, USA), the participating cytomorphologist accessed the PC and revised the BM cell classification digitally for their assigned samples. In addition, standard BM smears from the assigned patients were physically shipped to the participating cytomorphologists. The same investigators who performed the digital classification also performed the cell classification by using the conventional method.

To ensure unbiased results, patient clinical characteristics, sample identities between digital BM data and standard BM smears, and any data assigned to other participating cytomorphologists were anonymised. In addition, the study coordinators provided instructions for analysing the BM data unbiasedly (Text S3). Most of the participating cytomorphologists completed BM cell classification using both methods within 7–10 days.

### 2.5 Statistical Analysis

The correlation and comparability between the two BM classification methods were analysed using Pearson correlation analysis and the Bland–Altman method [21].

## 3 Results

### 3.1 Number and Success Rate of Digital BM Cell Classification

From January 2021 to June 2024, 775 BM aspirations were performed at MRTC Japan. The characteristics of the patients who underwent aspirations are summarised in Table 1. Many patients with MDS experience disease progression and undergo multiple BM examinations. Of the 775 aspirations, a portion resulted in dry taps.

**TABLE 1:** Characteristics of subjects.

Number of BM aspirations	775
Blasts (median and range, %)	6.2 (0–86.6)
Number of patients (Male/Female)	221 (147/74)
Age (median and range, years)	69 (17–97)
Initial diagnosis	
MDS	163
5q‐	2
SLD (with RS)	9 (1)
MLD (with RS)	49 (5)
EB‐1	60
EB‐2	36
EB‐F	7
MDS/MPN	6^a^
MPN	7^b^
AML	14
Lymphoid neoplasm	3^c^
Non‐clonal diseases	28^d^

Abbreviations: AML, acute myeloid leukaemia; EB, excess blasts, EB‐F, EB with fibrosis; MLD, multi LD; MPN, myeloproliferative neoplasm; RS, ringed sideroblasts; SLD, single lineage dysplasia.

^a^ Four patients with chronic myelomonocytic leukaemia and two with MDS/MPN, unclassifiable.

^b^ Three patients with essential thrombocythaemia and four with chronic myeloid leukaemia.

^c^ Each one patient with acute lymphoid leukaemia, chronic lymphoid leukaemia and T‐cell large granular lymphocyte leukaemia.

^d^ Ten patients with aplastic anaemia, eight with idiopathic cytopenia with undetermined significance (ICUS), two with pure red cell aplasia, two with thalassemia, two normal subjects and each one with immune thrombocytopenic purpura, cyclic neutropenia, familial Mediterranean fever and idiopathic erythrocytosis.

Successfully aspirated BM samples were subjected to both standard and CV smears (Figure S1). CV BM smears were prepared for 657 (96.2%) of the 683 aspirated samples; however, 26 samples were not prepared owing to clotting. The percentage of blasts in these 657 samples was less than 5% in 326 samples and 20% or more in 99 samples. Standard BM smears were analysed using conventional microscopy, which assessed the following: particle number, cellularity, rough estimation of increase/decrease of three cell classes (megakaryocytes [MKs], granulocytic cells and Ebls), morphology, status of rare cells (for example, macrophages) and presence of focal diseases (e.g., myeloma). The results of this analysis were compiled with the BM reports of the patients. In contrast, CV BM smears were used exclusively for BM cell classification, because various details that were obtained from standard BM smears, particularly regarding the status of particles, dysplasia, status of rare cells, are unavailable from the CV BM smears at present. The workflows from BM aspirations to the BM reports are summarised in Tables S1 and S2. The median time required by CellaVision to capture and pre‐classify BM cell images for a single smear was 10 min (range 7–21 min, based on the data from 100 consecutive smears obtained in January and February 2024). The processing time required was 15 min or more for nine smears, all of which originated from hypocellular BM samples. BM cell classification using digital images (pre‐classified by CellaVision and manually revised) was completed for all 657 samples.

### 3.2 Quality of Cell Images

**FIGURE 1:**
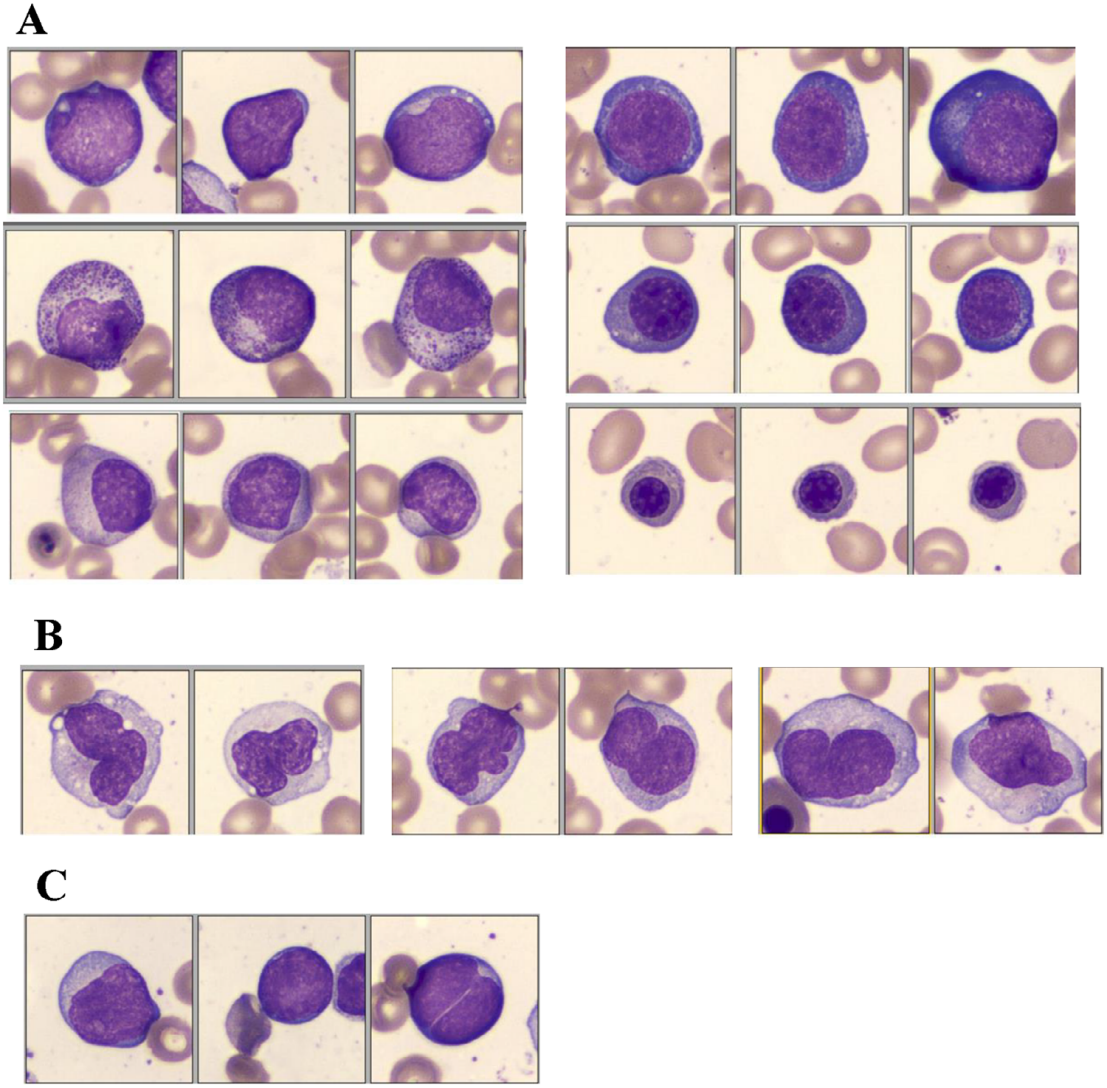
Representative examples of the classified digital images of BM cells. (A) Digital images from a 64‐year‐old male patient with MDS. The left panel shows myeloblasts (upper), promyelocytes (middle) and myelocytes (lower). The right panel shows proEbl (upper), basophilic Ebls (middle) and polychromatic Ebls (lower). (B) Digital images from a 64‐year‐old female patient with MDS. Monocytes (left), immature monocytes (middle) and promonocytes (right). (C) Digital images from a 69‐year‐old female patient with aplastic anaemia. A myeloblast (left) and haematogones (middle and right). Flow cytometric analysis in this case confirmed that 34% of CD34+ cells were CD19+ immature B cells (Figure S2).

**FIGURE 2:**
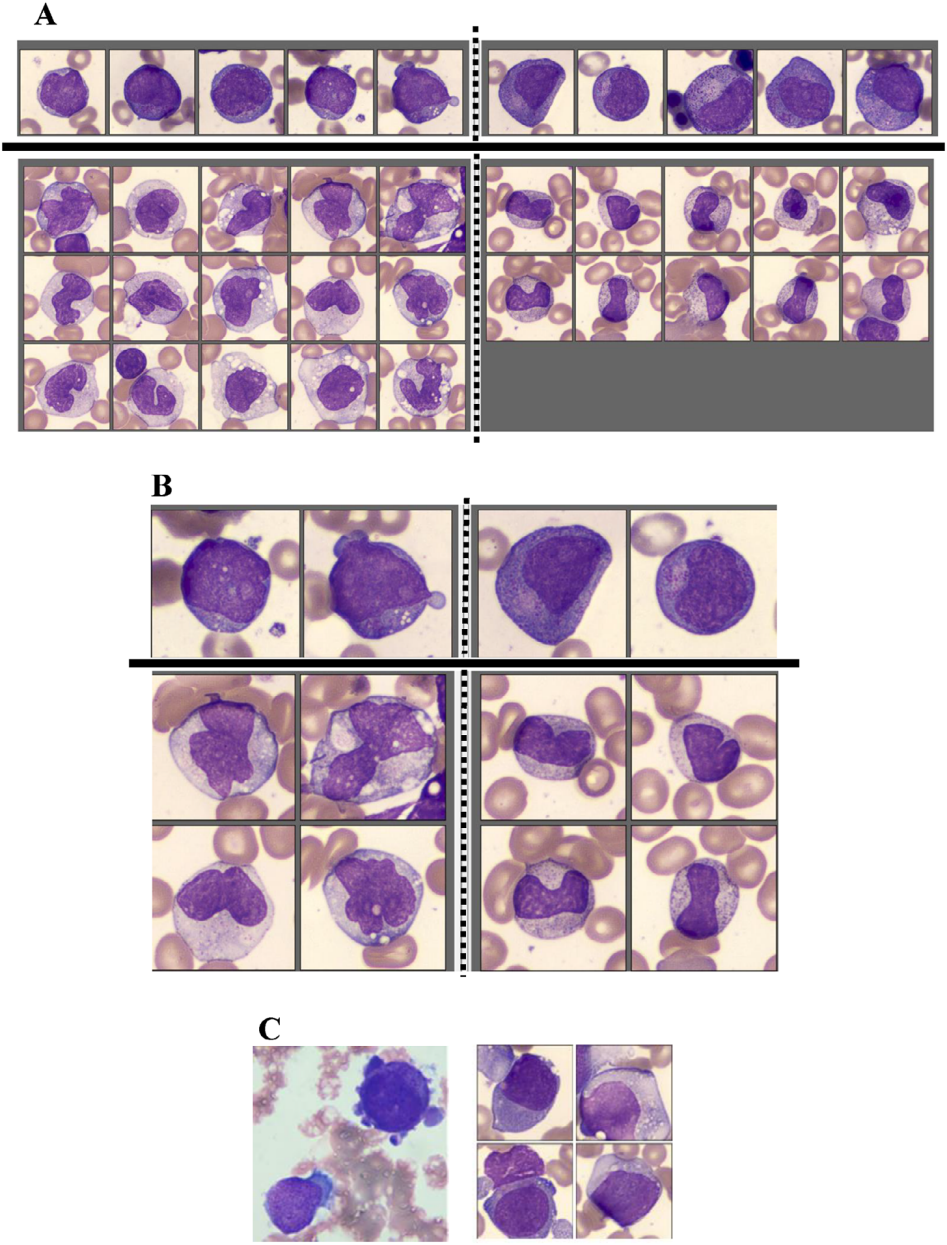
Other examples of the classified digital images of BM cells. (A) Side‐by‐side comparison of cell images in the same BM samples. The upper panels: the left shows myeloblasts (mostly Type II), and the right shows promyelocytes. Note the Golgi zones in promyelocytes. The lower panels: the left shows monocytes, and the right shows metamyelocytes. The cell classification can be revised easily, for example, by selecting the image and drag‐and‐drop to another cell fraction. (B) Cell images shown at the centre of panel A were enlarged. (C) Images from a 67‐year‐old female patient with MDS whose blasts showed megakaryoblastic immunophenotype. The left panel is microphotographs by conventional microscopy, in which blasts showed round nuclei and cytoplasmic blebs. These morphologic features were obscure in digital images from the same BM sample (the right panel). Flow cytometry data of this case are shown in Figure S2.

Because digital images were captured at 2000 × magnification and analysed on a large high‐quality screen, morphological details were easier to examine than with conventional microscopy. This advantage was particularly evident in identifying Mbls, haematogones, various monocytic cells [22] and the distinction between monocytic cells and dysplastic (particularly hypogranular) neutrophilic cells (Figure 1A–C). In addition, cell images can be compared side‐by‐side between cell classes and enlarged on‐screen, which makes cell classification more precise and reliable than conventional microscopy (Figure 2A–C). A representative example of all cell images from a BM sample is provided in Figure S3. However, because CV BM smears were prepared from anticoagulated samples and were more widely spread than conventional BM smears, the cell morphology was not identical between the two types of smears. Occasionally, clear morphological differences were observed between the two groups (Figure 2C).

### 3.3 Concordance in the BM Cell Classification Data Between the Digital and Conventional Methods

**FIGURE 3:**
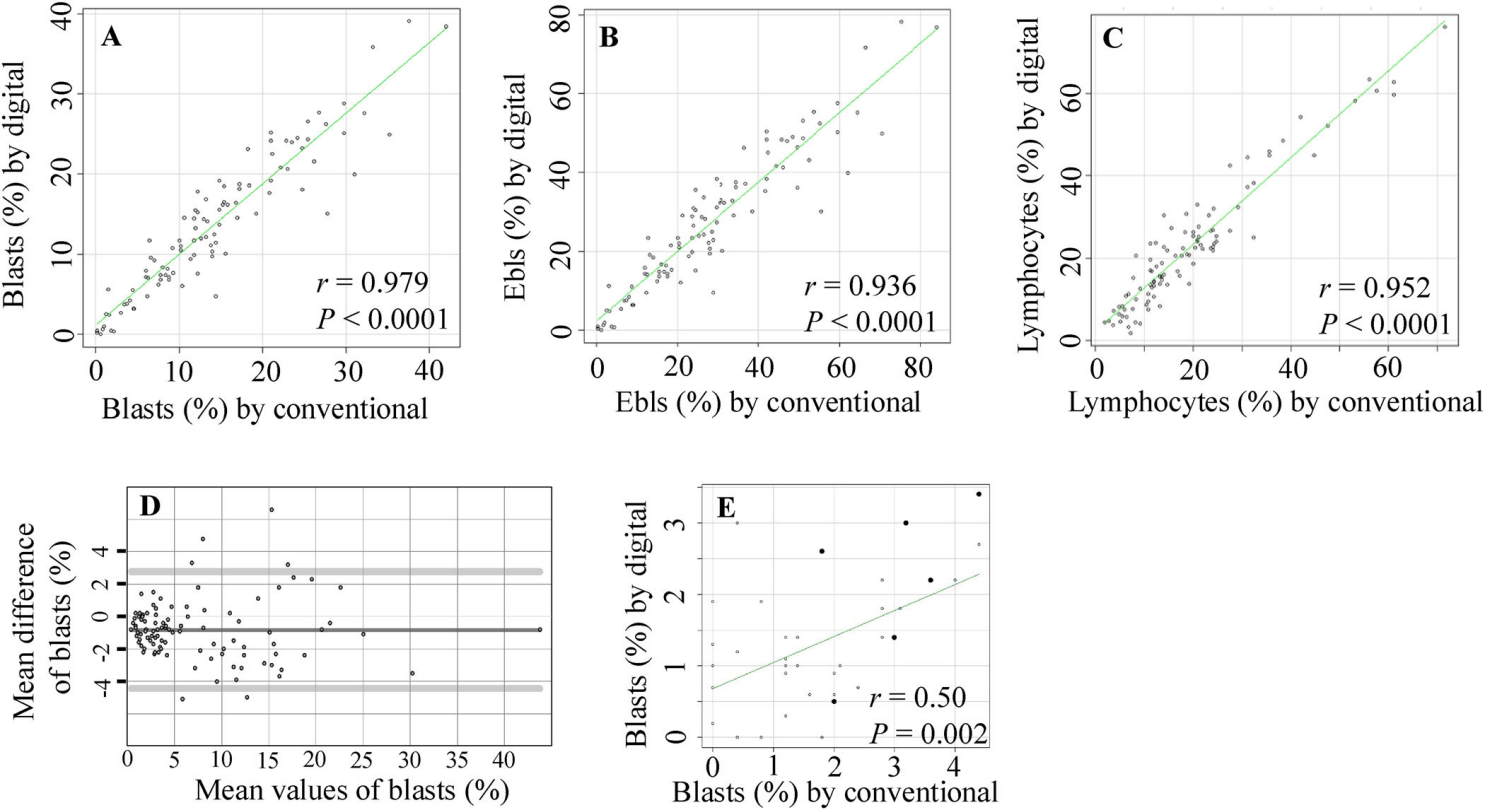
Concordance in the BM cell classification data determined by the digital and conventional methods. (A–C) The 100 data sets from patients with MDS and leukaemic MDS. The correlation between the paired data (percentages), which were determined by the two methods for blasts (A), Ebls (B), and lymphocytes (C), were shown. (D) The paired data for blasts in Panel A were analysed by the Bland–Altman method. (E) The data from 37 samples from non‐clonal diseases (open circles) and MPNs (closed circles). The correlation between the blast percentages, which were determined by the two methods, was shown.

First, we analysed the correlation between the percentages of cells determined by the digital and conventional methods at MRTC Japan. A strong correlation was observed in 100 MDS samples; the data in representative cell classes are shown in Figure 3A–C and data from other cell classes are shown in Figure S4. When the percentage of blasts in these MDS samples was analysed using the Bland–Altman method; the percentage of blasts was, on average, 0.84% lower using the digital method than with the conventional method, and the limits of agreement between the two methods were +2.77 to −4.46% (Figure 3D). Analysis using logistic transformation data showed that the mean difference did not increase with the mean blast count (data not shown). To further examine the clinical significance of the differences between the two methods, MDS subgroups (i.e., low‐grade, EB‐1, EB‐2 and leukaemic) were determined using the same paired data on blasts. The MDS subgroups were concordant in 86 of the 100 MDS cases, and the only disagreements in diagnoses were with adjacent categories, demonstrating acceptable reproducibility between the two analysis methods for MDS classification (Table 2). Even among the 14 cases with inconsistent results, the difference in blast percentages between the two methods was low in most instances (Table 2) (see Text S4 for more information regarding discordant diagnoses). As expected, the correlation between the paired blast data from the digital and conventional methods was poor for the 37 samples from non‐clonal conditions and MPNs; however, all blast data for both methods were < 5% in all of these samples (Figure 3E).

**TABLE 2:** Concordance of MDS categories determined by the digital and conventional methods.

		Diagnoses by data from the conventional method			
		Low‐grade	EB‐1	EB‐2	Leukaemic
Diagnoses by data from the digital method	Low‐grade	51	3^b^	0	0
	EB‐1	1^a^	10	7^d^	0
	EB‐2	0	1^c^	19	1^f^
	Leukemic	0	0	1^e^	6

*Note*: The data are numbers of patients. The weighted kappa value is 0.93198 (95% confident interval 0.89595–0.96801). The difference in blast percentages between the two methods in patients showing unconcordant diagnoses were 0.6% (one case in a), 1%, 2.4% and 5.1% (three cases in b), 4.8% (one case in c), 1.7%, 2%, 2.3%, 2.6%, 3.1%, 3.9% and 4% (seven cases in d), 2.3% (one case in e) and 2.4% (one case in f).

Finally, the percentages of blasts determined in the multicentre study were examined (Table 3). Although all the participants were first‐time users of digital BM cell classification, their results were acceptably concordant across most samples. In the four samples, the blast percentage difference between the two methods was greater than five and was associated with diagnostic discrepancies. However, when these data were compared with the MRTC Japan data, the results obtained via the digital method were closer to the MRTC reference data for three of the four samples (Table 3; see Text S5 for details).

**TABLE 3:** Blast percentages determined in the multicentre study.

		Samples to each examiner					
Examiner	Methods	1	2	3	4	5	6
1	Digital	3.2	5.0	7.0	9.5	12.4	18.8
	Conventional	2.2	3.6	7.2	9.1	10.8	22.2
2	Digital	0.3	5.1	5.1	8.8	10.1	14.2
	Conventional	2.0	4.4	8.0	7.8	7.9	14.2
3	Digital	1.2	1.6	3.9	7.0	9.2^a^	
	Conventional	1.4	5.5	5.8	5.0	15.4	
4	Digital	3.6	9.6	17.0	19.4	29.6	
	Conventional	1.8	5.5	13.0	15.4	32.4	
5	Digital	3.4	4.2	8.1	18.7	32.4	
	Conventional	4.3	4.1	12.3	21.5	26.6	
6	Digital	4.0^b^	7.9	12.0	22.9	29.6	
	Conventional	10.0	10.0	15.0	22.0	22.0	
7	Digital	1.2	2.1	6.0	15.2^c^	24.7^d^	
	Conventional	0.8	0.5	3.3	7.0	5.5	

*Note*: The sample identity between the standard BM smears and digital BM data were blinded to the examiners and the overlap of samples between examiners was minimal. Blast percentages determined by the digital and conventional methods at the MRTC Japan were 17.6 and 20 (a), 3.7 and 4.4 (b), 18.8 and 16.4 (c), 23.5 and 21.7 (d).

## 4 Discussion

The cytomorphological examination of BM cells is essential for diagnosing numerous diseases. However, mastering this skill requires a substantial amount of time and an environment [10]. In addition, even after achieving expertise, cytomorphological examination remains effort‐intensive and cannot eliminate subjectivity [5, 6, 7, 8, 9]. In this study, we introduce a machine‐assisted, human‐centred digital method for BM cell classification. The data, particularly blast count in MDS samples, that were obtained with the digital and conventional methods were concordant, and even first‐time users (cytomorphologists) accurately classified cell using this approach. MRTC Japan uses this digital method as a supplementary differential count method to complement the standard optical microscopic examination because other information that is obtained from standard BM smears, particularly regarding the status of particles, dysplasia, status of rare cells, are currently unavailable from CV BM smears.

Our method has the following advantages: softens intensive labour (operators can classify BM cells in spare time using a large PC screen, with the ability to pause and resume the classification anytime), provides objectivity (anyone can confirm and revise the results which are saved on a PC, and this facilitates teaching and professional development), and is more user‐friendly for the digital era, facilitating broader adoption of cytomorphology (see Text S6 and Table S3 for details). Preserving cytomorphological skills in haematology is mandatory. One might think that more sophisticated diagnostic technologies may be developed in the future. However, even to develop such tools, samples from patients who are correctly diagnosed using various techniques, in which cytomorphological data often play a central role, are needed (see Text S7 for more details). Recent articles have reported that BM cell sampling is problematic in real‐world practice [11, 12]. We believe that the cytomorphological skills are linked to the BM sampling skills. Clinicians with essential cytomorphological skills can recognise the problem when they encounter inadequate BM samples, such as aspicular BM smears. In addition, such individuals may be motivated to obtain and examine BM cells independently, which helps preserve essential haematology skills.

Some caveats exist when the proposed method is applied. First, a small volume of spicular BM sample should be obtained and processed quickly. Although EDTA‐2K is a common reagent used for blood cell analysis, excess EDTA‐2K changes cell morphology. Therefore, we used a mini tube designed with a small sample volume. Moreover, after 2 h, the EDTA‐2K anticoagulated haematopoietic cells may have lost their original morphology [23, 24]. CV BM smears were stained within 1 h (usually within 30 min) of BM aspiration. Nevertheless, we occasionally observed a clear difference in cell morphology between the standard and CV BM smears. To overcome this problem and ensure the reliability of digital data, it is important to use conventional BM smears (Figure S1). The CellaVision program, designed for classifying PB cells, currently induces errors in classifying BM cells and cannot analyse the dysplasia that defines MDS. AI‐based digital methods are attractive options for solving these problems, and further studies are required to achieve this goal (see Text S8 for details). Second, in standard microscopy, the cellular trails behind the particles, where PB dilution is minimal, are recommended for cell classification [25, 26]. CV technologies capture cell images randomly without specific area selection. Considering this difference, anticoagulated BM samples were mixed using a dropper with a relatively large bore size to disaggregate the cells packed in the BM particles. This bore size is an important factor. When a pipette with a smaller bore size, such as a disposable micropipette, is used, the BM particles become clogged at the tip and fail to disaggregate, leading to unreliable data. Third, it is ideal for each digital image to contain a single nucleated cell with preserved morphology (Method S1). To achieve this, well‐spread smears, created using samples with optimal cell concentrations are required. Therefore, undiluted and diluted BM samples were used to prepare CV BM smears. Fourth, data concordance between digital and conventional methods should be thoroughly examined in each institution before clinical use.

Most BM samples analysed in MRTC Japan were obtained from patients with MDS or conditions requiring differentiation from MDS. Therefore, the applicability of our method for the analysis of other diseases remains to be verified. In particular, it is assumed that the present method may not be suitable for diagnosing focal diseases such as multiple myeloma and metastatic neoplasms, including lymphoma. For BM samples with monotonous neoplastic cells, BM cell classification is usually required for 200–300 cells and is not laborious. Meanwhile, BM cell classification for samples from patients with MDS and suspected MDS requires the classification of 500 cells and is a laborious task. This labour becomes more intense when the BM is hypocellular. In this regard, the present method is particularly helpful for analysing such BM samples.

In summary, this study introduced a machine‐assisted, human‐centred BM classification method. This method softens intensive labour, enhances objectivity in the current BM cell classification work, and helps to preserve cytomorphological skills which are essential in haematology [10].

## Author Contributions

Designed the study, analysed the data and wrote the manuscript: Kiyoyuki Ogata and Yumi Yamamoto. Performed the experiments: Kiyoyuki Ogata, Yuto Mochimaru, Leonie Saft, Gerrit Sijm, Bouchra Badaoui, Canan Alhan, Yosuke Kato, Daisuke Sakamoto, Naoko Chiba, Naho Murata, Nana Kasai and Nicolas Chapuis. Collected the data: Nana Kasai, Naoya Kawahara and Mika Ogata. Contributed to the study and reviewed the data: Canan Alhan, Orianne Wagner‐Ballon and Arjan van de Loosdrecht. All authors contributed to the interpretation of the results, preparation and review of the manuscript and approval of the final version of the manuscript.

## Funding

The authors have nothing to report.

## Ethics Statement

This study was approved by the Institutional Review Board of MRTC Japan (IRB189), and the procedures were performed in accordance with the Helsinki Declaration.

## Consent

Written informed consent was obtained from all the patients.

## Conflicts of Interest

The authors declare no conflicts of interest.

## Supporting information




**Supporting File 1**: jha270205‐sup‐0001‐figureS1.pdf

**Supporting File 2**: jha270205‐sup‐0002‐figureS3.pdf

**Supporting File 3**: jha270205‐sup‐0003‐figureS3.pdf

**Supporting File 4**: jha270205‐sup‐0004‐figureS4.pdf

**Supporting File 5**: jha270205‐sup‐0005‐tableS1.xlsx

**Supporting File 6**: jha270205‐sup‐0006‐tableS2.xlsx

**Supporting File 7**: jha270205‐sup‐0007‐tableS3.xlsx

**Supporting File 8**: jha270205‐sup‐0008‐SuppMat.docx

## Data Availability

The data that support the findings of this study are available from the corresponding author upon reasonable request.
