# Supplementary material for: Development of Machine‐Assisted, Human‐Centred Bone Marrow Cell Classification: Feasibility Analysis in Patients With Myelodysplastic Syndromes
Source: EJHaem. 2025 Dec 16;6(6):e70205. doi: 10.1002/jha2.70205 (PMC12707303; doi:10.1002/jha2.70205)
Supplement: Supplementary file 1 — Supporting File 1: jha270205‐sup‐0001‐figureS1.pdf [file JHA2-6-e70205-s003.pdf]

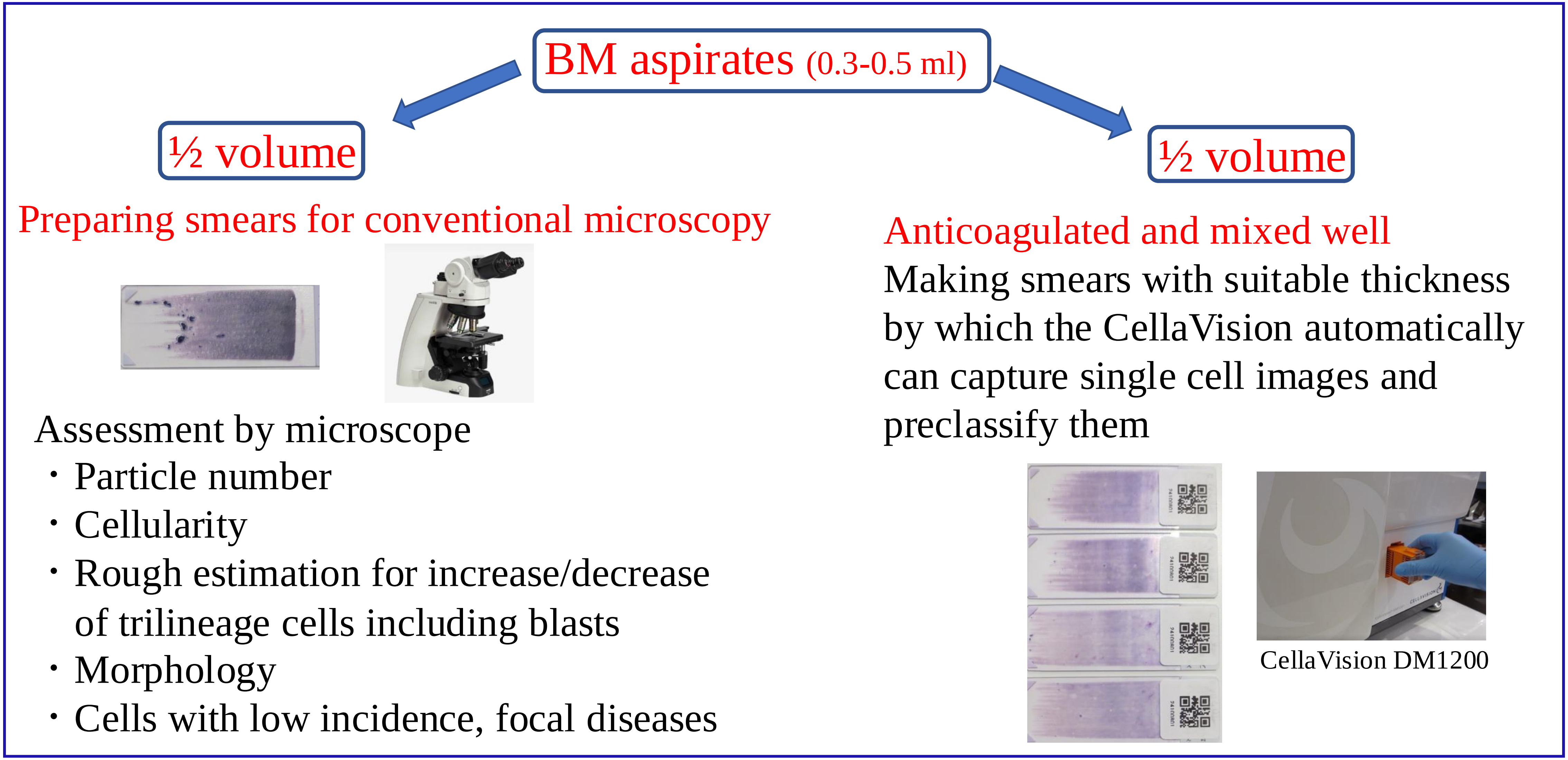

Supplementary Figure 1. BM cell sample processing and capturing cell images by the CellaVision.
