# Supplementary material for: Development of Machine‐Assisted, Human‐Centred Bone Marrow Cell Classification: Feasibility Analysis in Patients With Myelodysplastic Syndromes
Source: EJHaem. 2025 Dec 16;6(6):e70205. doi: 10.1002/jha2.70205 (PMC12707303; doi:10.1002/jha2.70205)
Supplement: Supplementary file 2 — Supporting File 2: jha270205‐sup‐0002‐figureS3.pdf [file JHA2-6-e70205-s008.pdf]

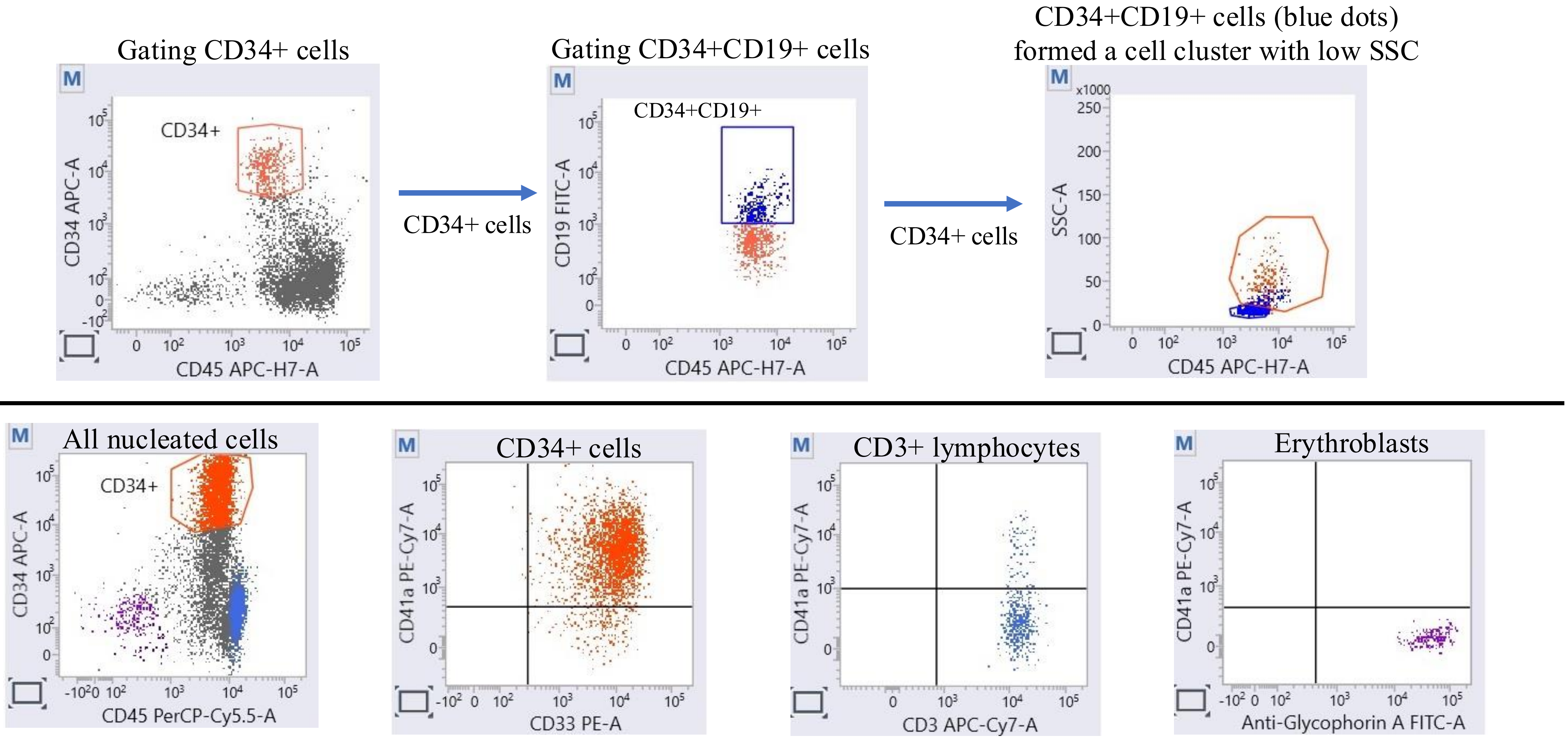

Supplementary Figure 2. Flow cytometry (FCM) data. **Upper panel**: The case is shown in panel C of Figure 1. Thirty-four percent of CD34+ cells co-expressed CD19 and formed a typical cell cluster with low side scatter (SSC) among CD34+ cells on a CD45 versus SSC plot, corresponding to stage I haematogones. **Lower panel:** The case is shown in panel C of Figure 2. Most CD34+ cells were positive for CD41 and CD33, while other cell fractions were negative for CD41 (see reference 18 for method details to rule out the false positivity of CD41).
