# Supplementary material for: Development of Machine‐Assisted, Human‐Centred Bone Marrow Cell Classification: Feasibility Analysis in Patients With Myelodysplastic Syndromes
Source: EJHaem. 2025 Dec 16;6(6):e70205. doi: 10.1002/jha2.70205 (PMC12707303; doi:10.1002/jha2.70205)
Supplement: Supplementary file 3 — Supporting File 3: jha270205‐sup‐0003‐figureS3.pdf [file JHA2-6-e70205-s001.pdf]

Blasts

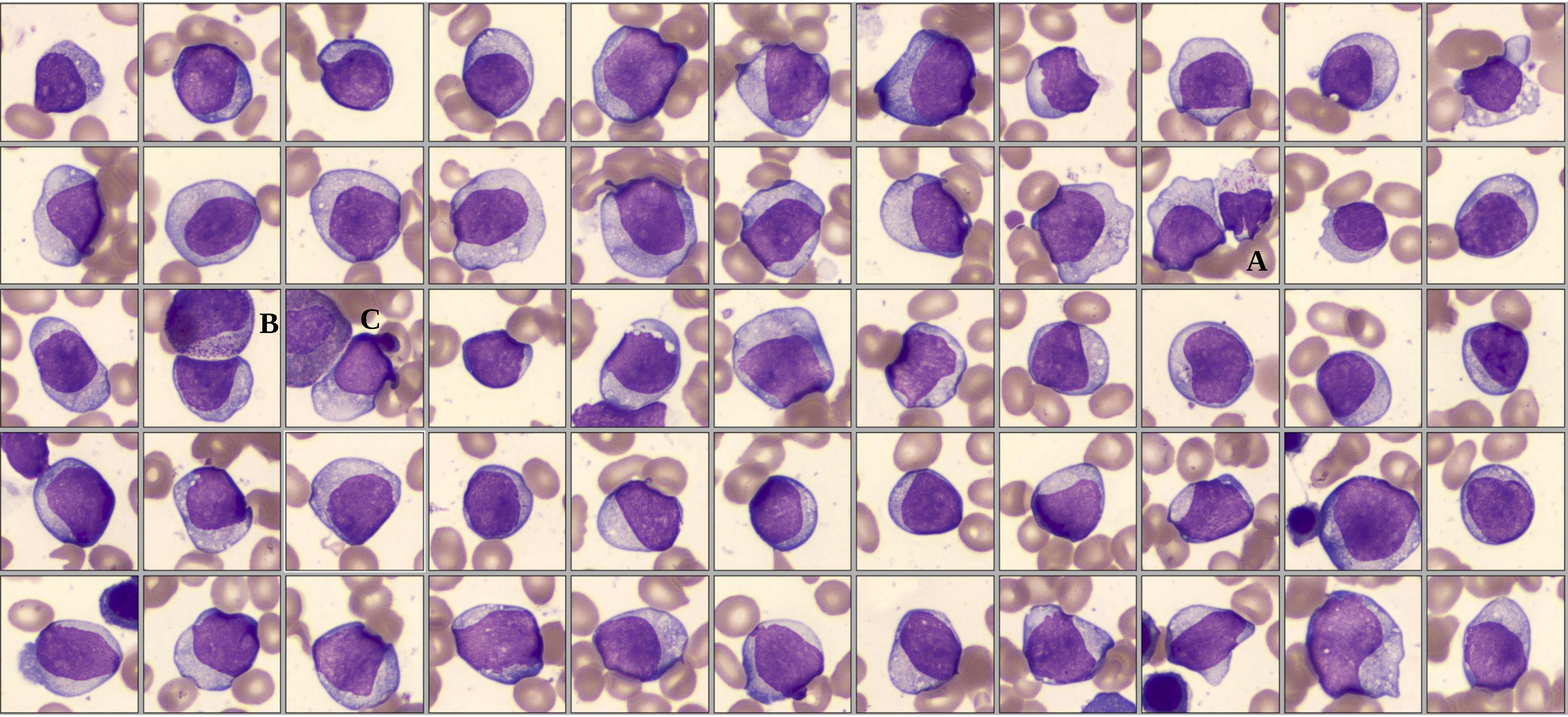

Blasts

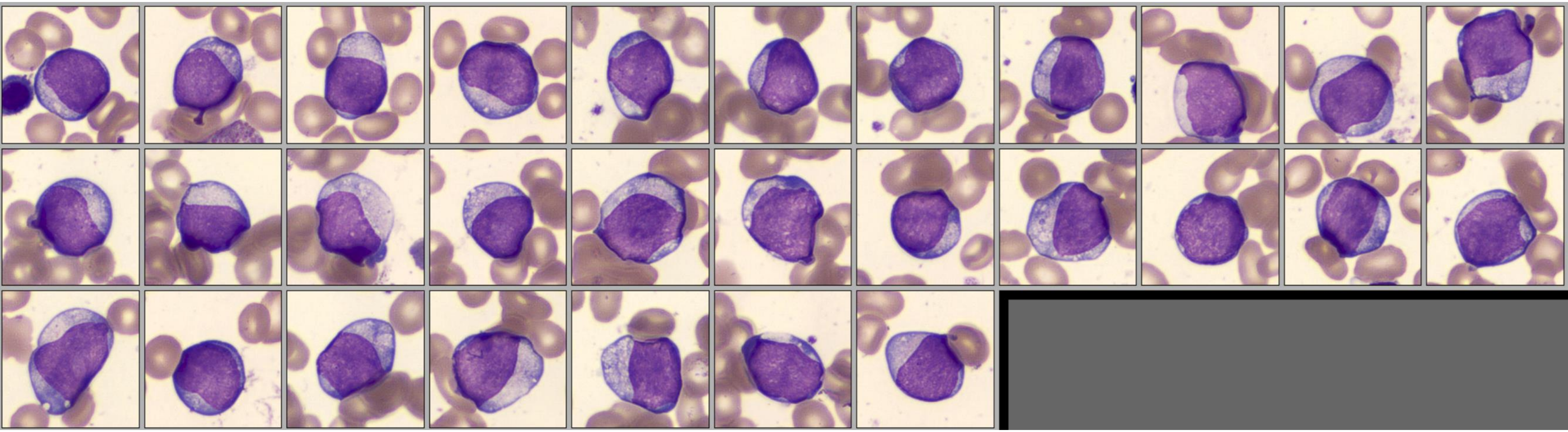

Promyelocytes

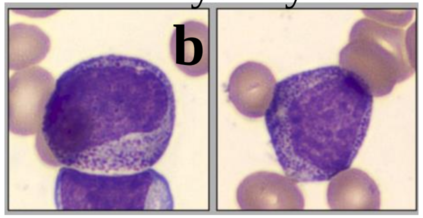

Myelocytes

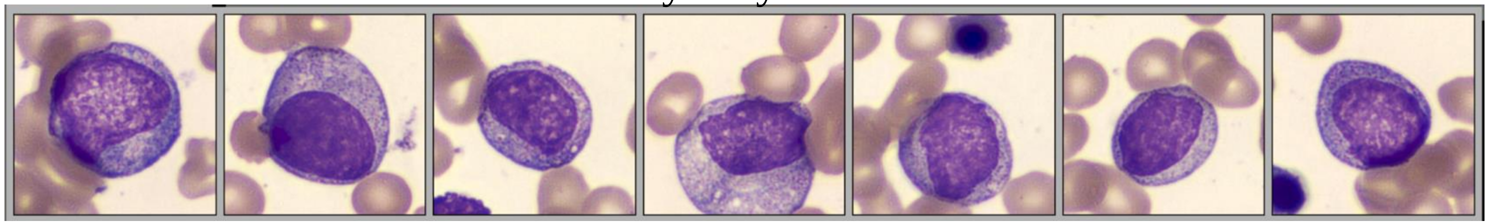

Metamyelocytes

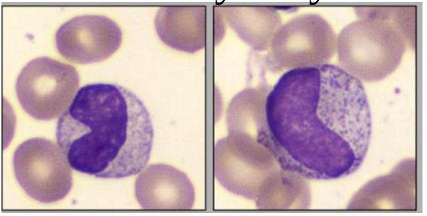

Band neutrophils

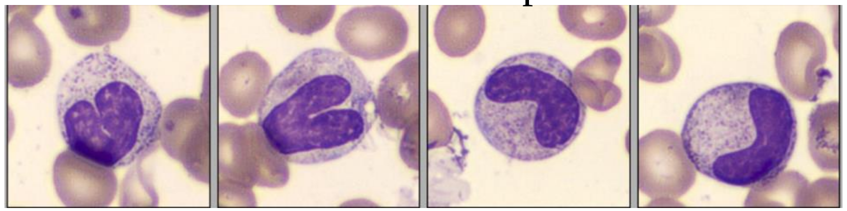

Segmented neutrophils

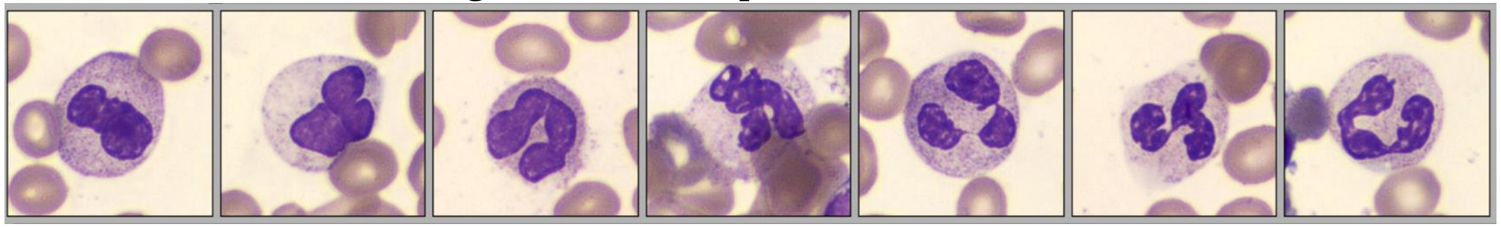

Eosinophils

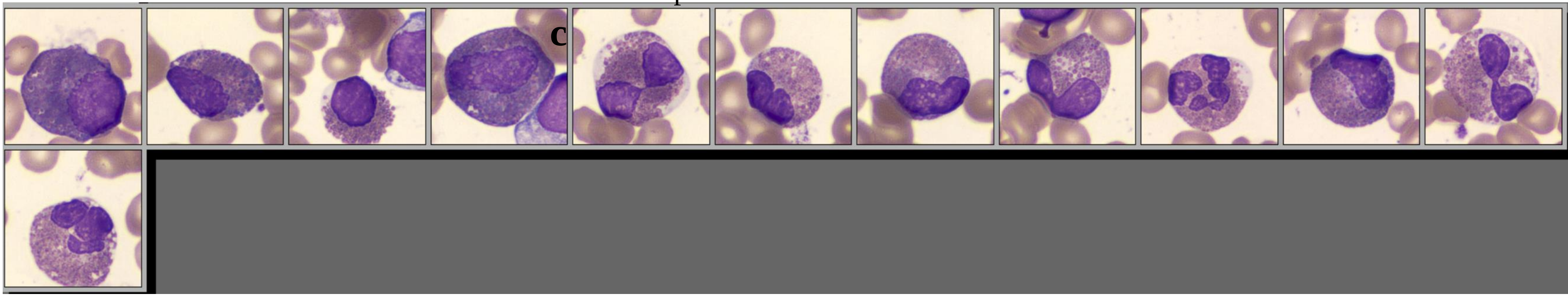

Basophil

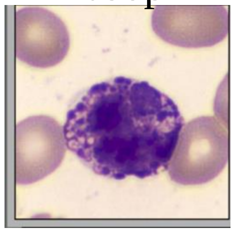

Monocytes

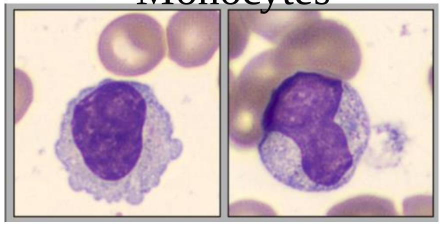

Macrophage

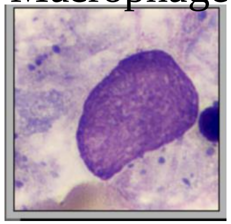

Plasma cells

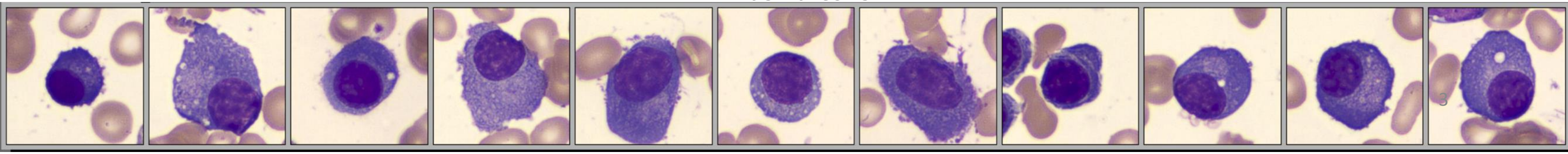

Lymphocytes

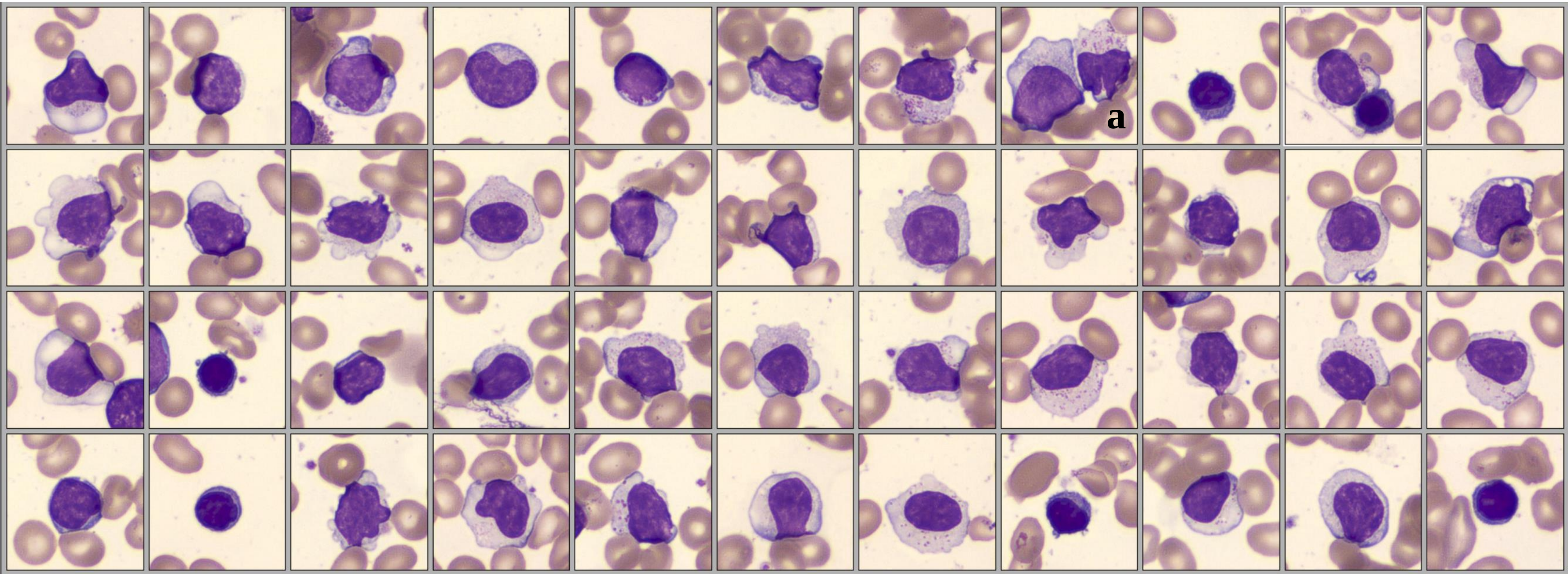

Lymphocytes

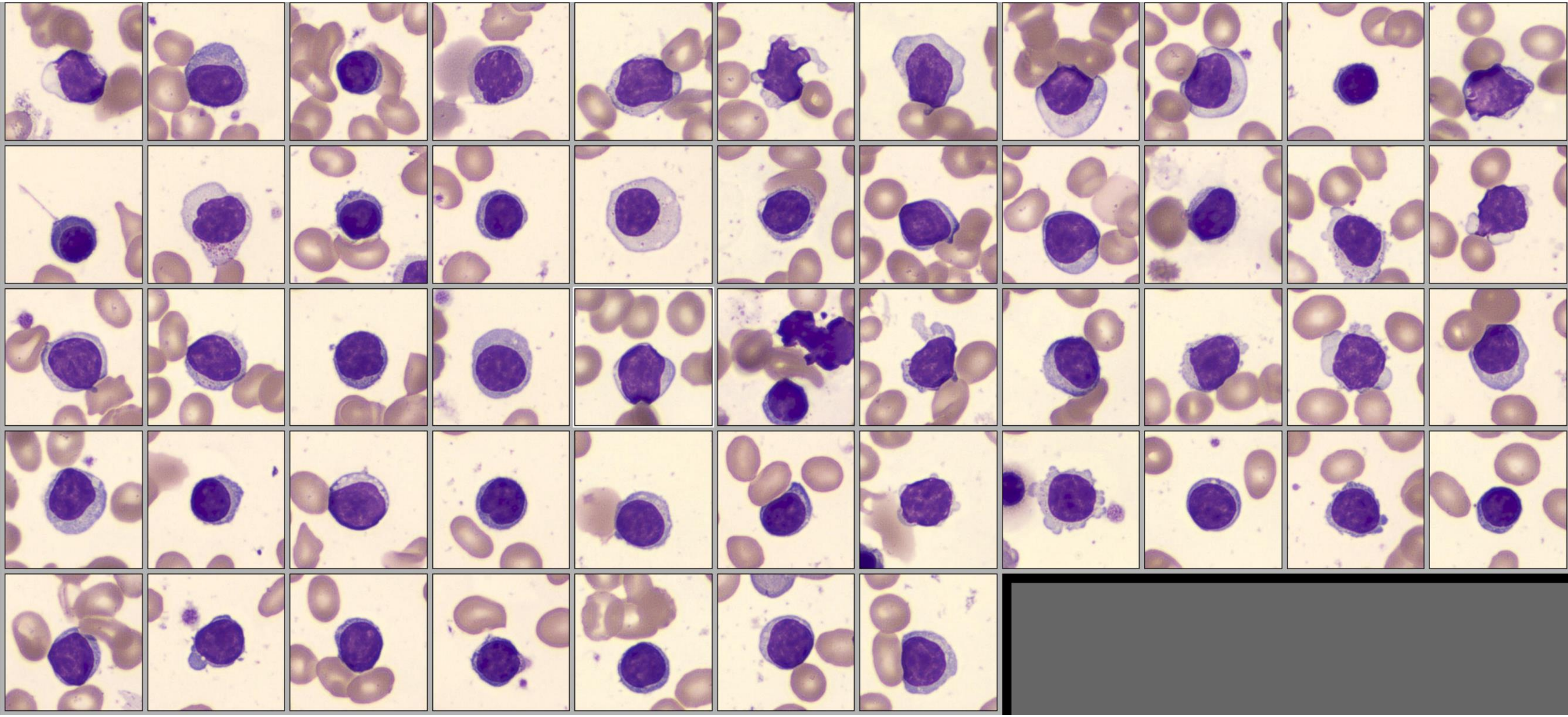

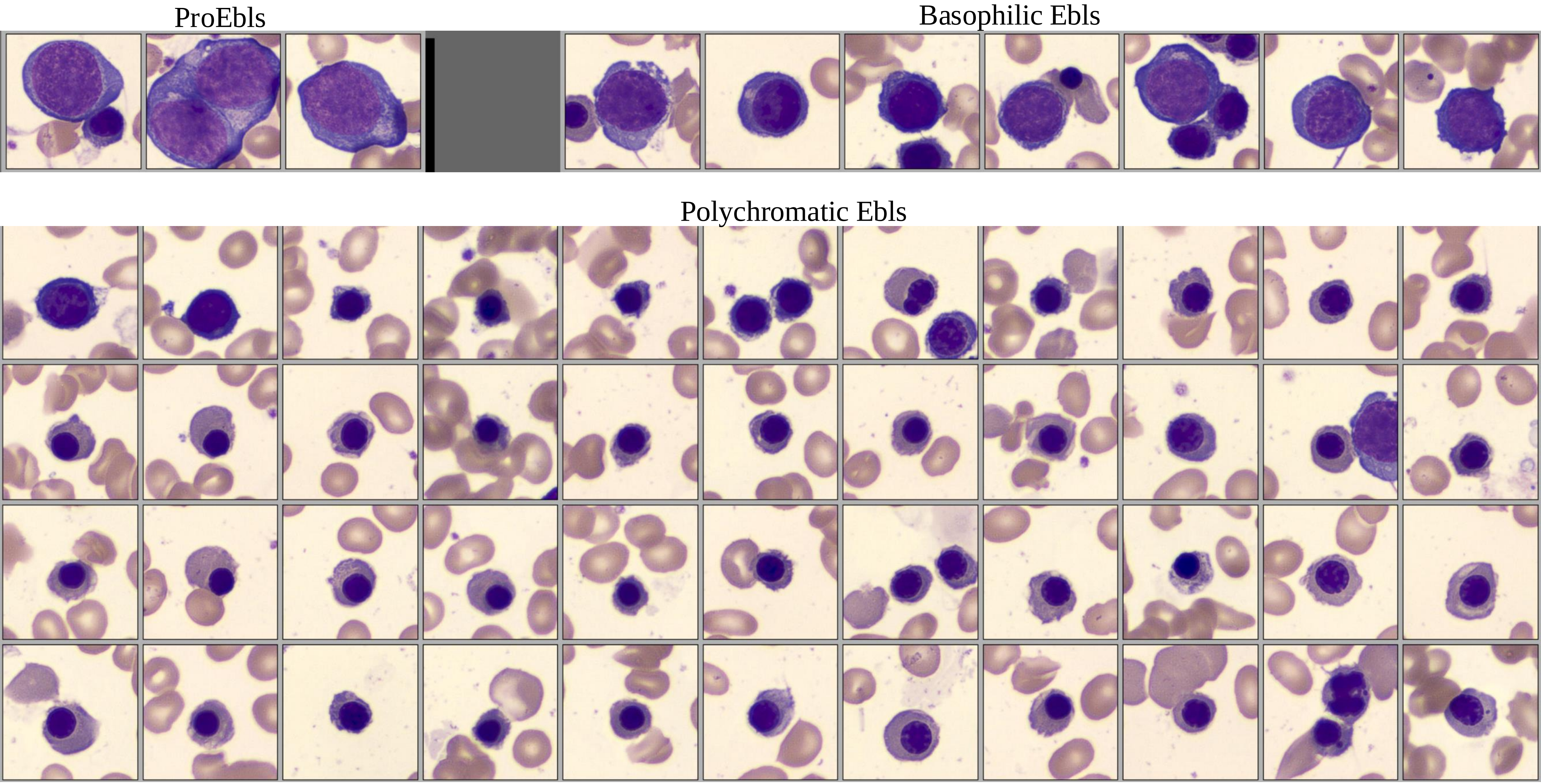
ProEbls
Basophilic Ebls
Polychromatic Ebls

Polychromatic Ebls

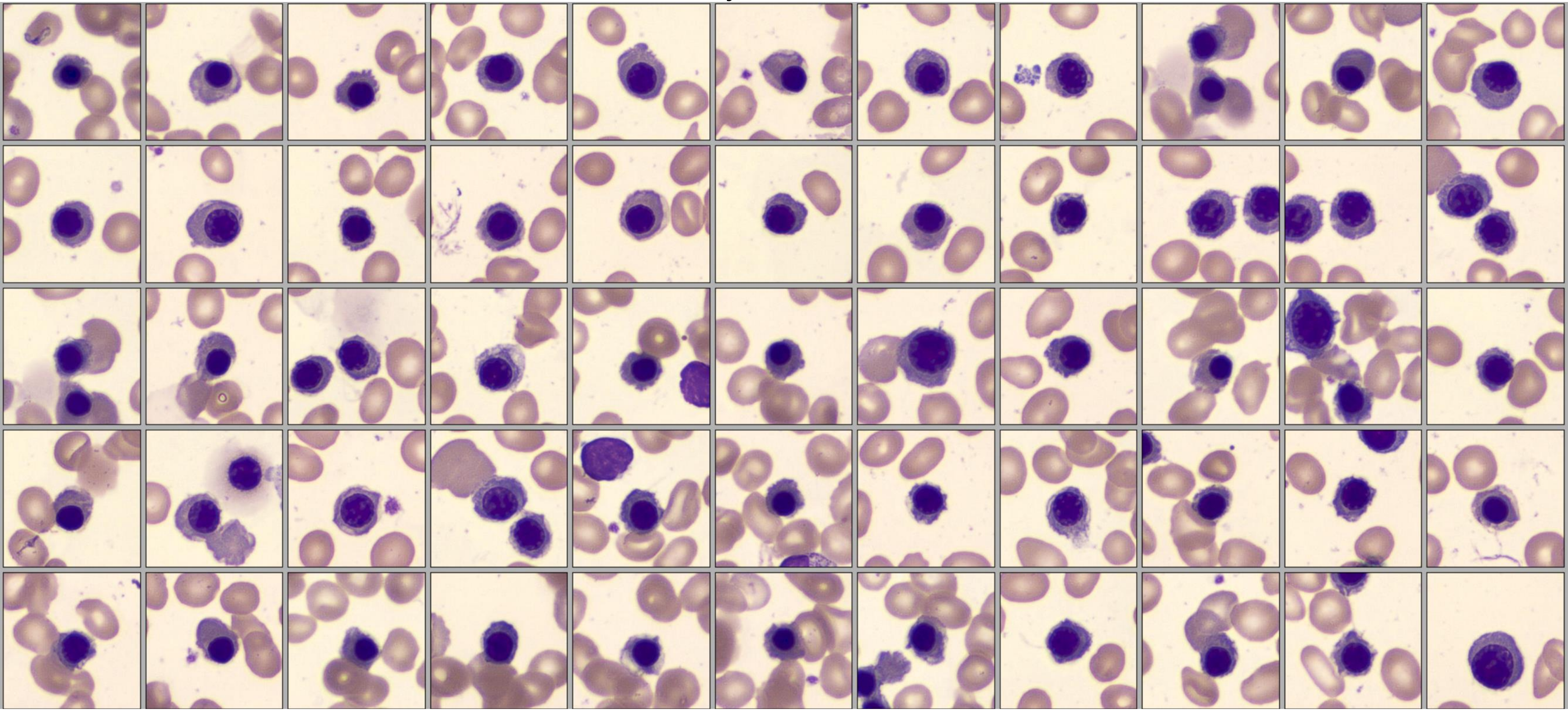

Polychromatic Ebls

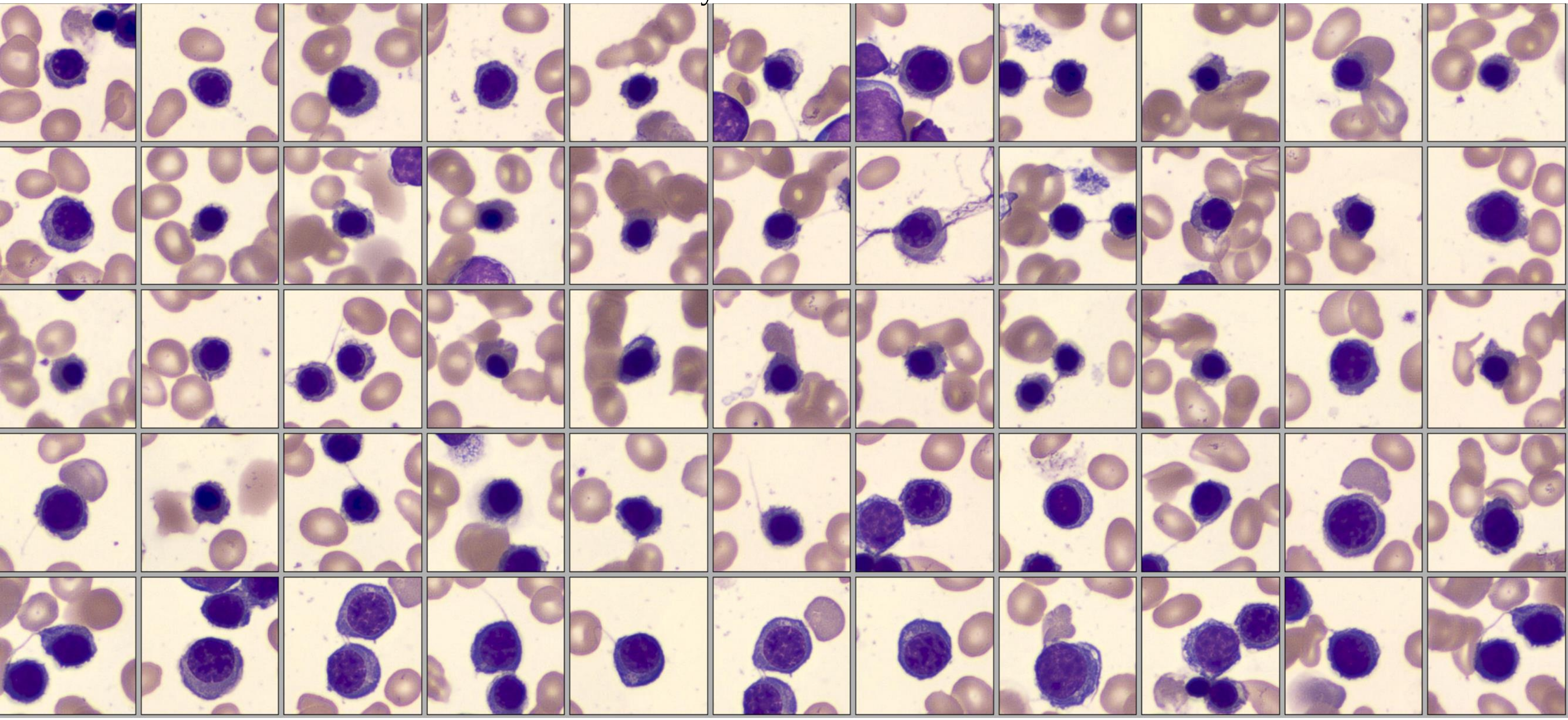

Polychromatic Ebls

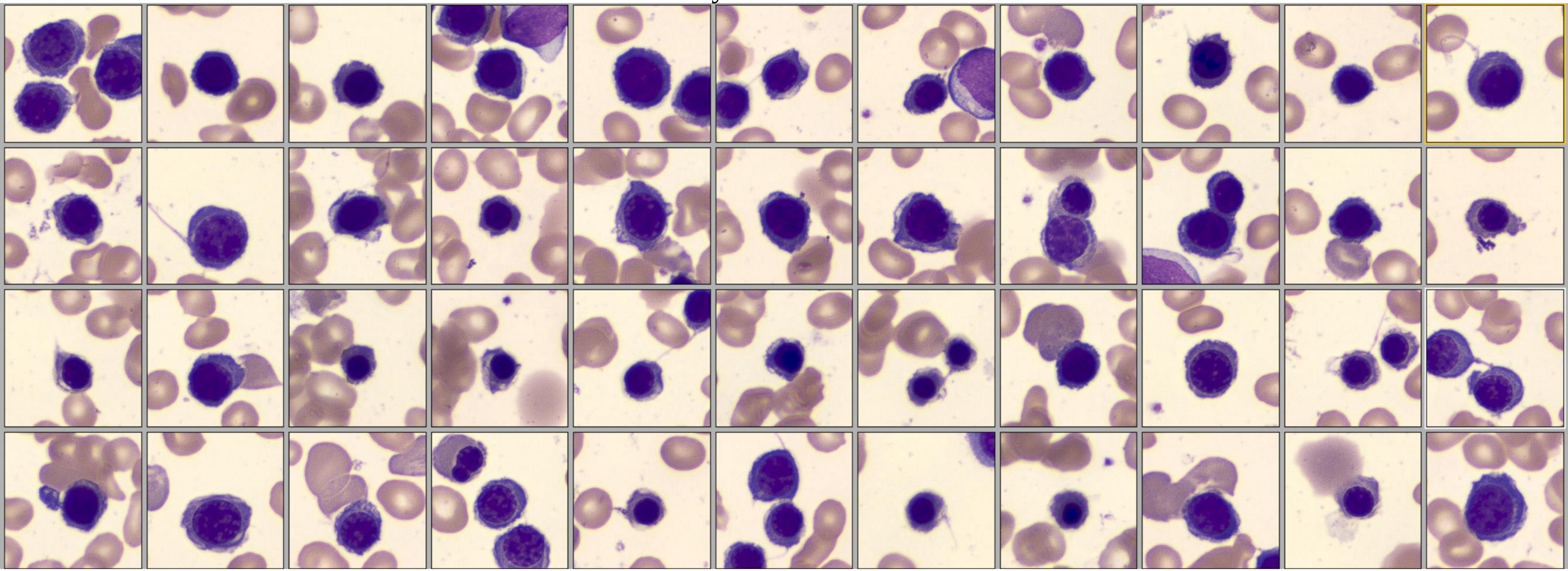

Polychromatic Ebls

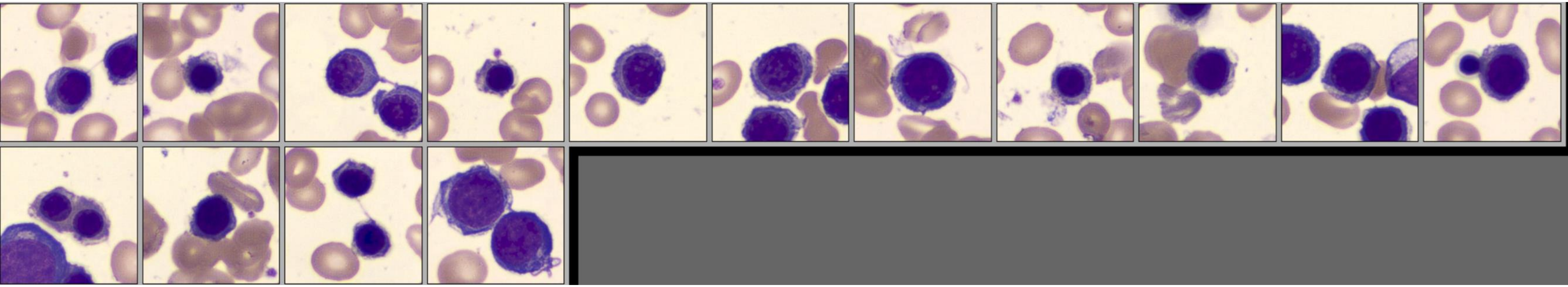

Orthochromatic Ebls

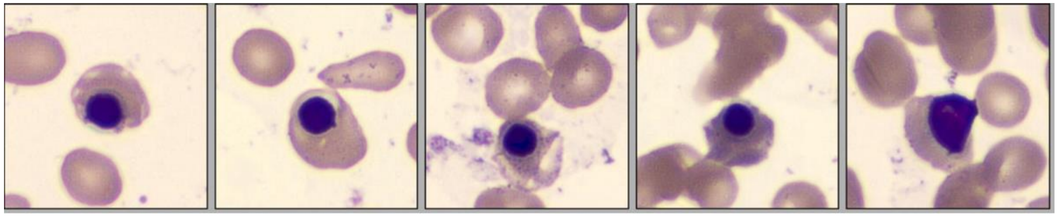

Supplementary Figure 3. Cell images from a 78-year-old male patient with EB-2. The CellaVision captured 755 images from one BM smear. We deleted 328 images which did not contain cells to be classified (the vast majority were smudge cells, and others include platelet aggregations, giant platelets, etc.), and the remaining 456 images are shown. Three images classified in the blast fraction contained a lymphocyte, a promyelocyte and an eosinophil, respectively, alongside blasts (marked as A–C on page 1). These three cells were classified into the respective cell fractions (marked as a–c on pages 2–4). For seeing clear images, it is preferable to use a display with a similar or better resolution applied in this study (3820 x 2160 UHD).
