# Supplementary material for: Development of Machine‐Assisted, Human‐Centred Bone Marrow Cell Classification: Feasibility Analysis in Patients With Myelodysplastic Syndromes
Source: EJHaem. 2025 Dec 16;6(6):e70205. doi: 10.1002/jha2.70205 (PMC12707303; doi:10.1002/jha2.70205)
Supplement: Supplementary file 4 — Supporting File 4: jha270205‐sup‐0004‐figureS4.pdf [file JHA2-6-e70205-s006.docx]

**Supplementary Figure 4.** Concordance in the BM cell classification data determined by the digital and conventional methods. The 100 data sets

from patients with MDS and leukemic MDS. The X- and Y-axes show the percentages that were determined by the conventional and digital methods, respectively.


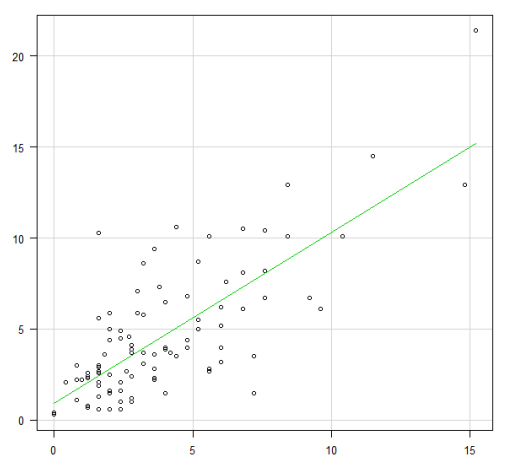


Monocytes

*r* = 0.766

*P* < 0.001


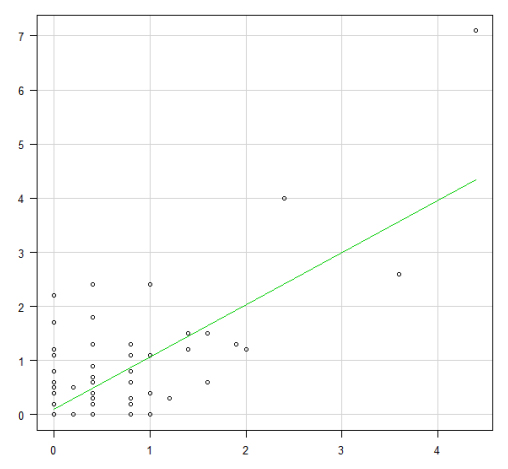


Basophils

*r* = 0.723

*P* < 0.001


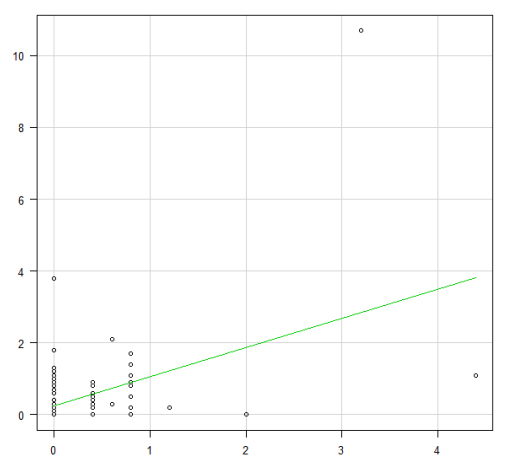


Promonocytes and

immature monocytes

*r* = 0.443

*P* < 0.001


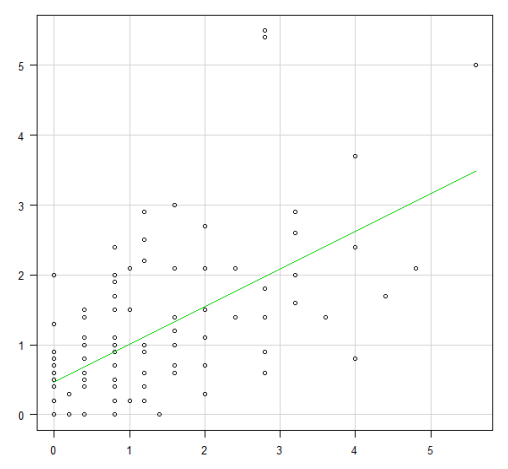


Plasma cells

*r* = 0.593

*P* < 0.001


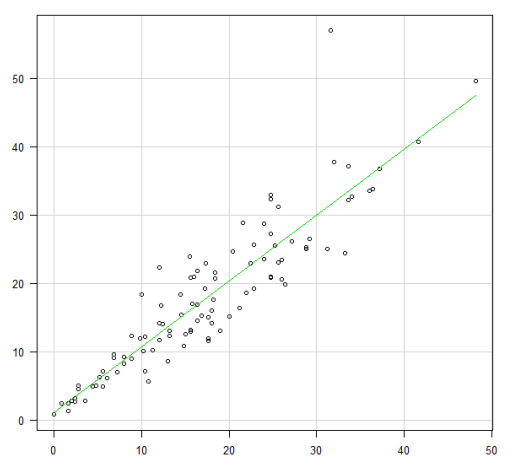


Band and segmented neutrophils

*r* = 0.910

*P* < 0.001


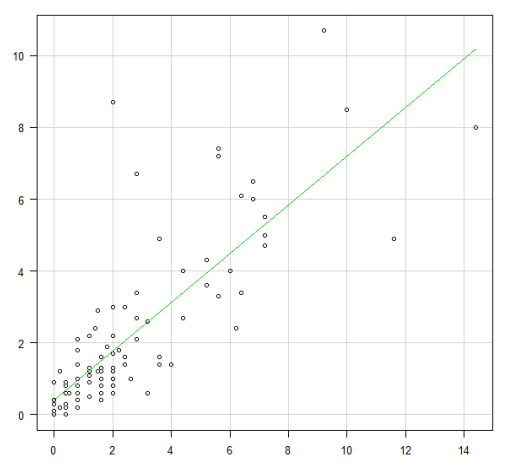


Eosinophils

*r* = 0.808

*P* < 0.001


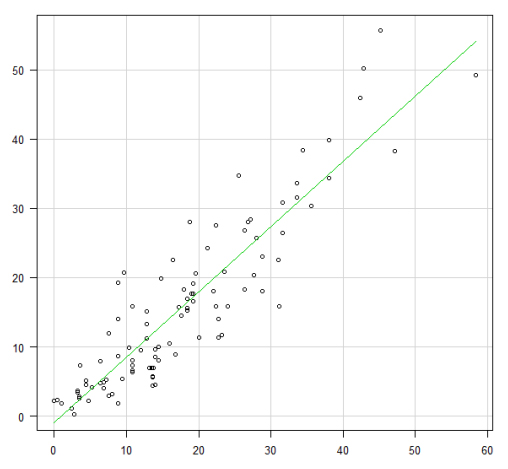


Promyelocytes, myelocytes, and metamyelocytes

*r* = 0.905

*P* < 0.001
