# Supplementary material for: Development of Machine‐Assisted, Human‐Centred Bone Marrow Cell Classification: Feasibility Analysis in Patients With Myelodysplastic Syndromes
Source: EJHaem. 2025 Dec 16;6(6):e70205. doi: 10.1002/jha2.70205 (PMC12707303; doi:10.1002/jha2.70205)
Supplement: Supplementary file 8 — Supporting File 8: jha270205‐sup‐0008‐SuppMat.docx [file JHA2-6-e70205-s004.docx]

**Supplementary Texts**

**Supplementary Text 1 (Method)**

The reason for the criterion that most of each image contained one nucleated cell was as follows. Because CellaVision interprets one image as a single cell, additional work must be performed if one image contains multiple nucleated cells. Examples are as follows: 1) If one image contains two cells, for example, a promyelocyte and a myelocyte, the CellaVision classifies this image into “Unclassifiable” or other folders. In this case, we had to duplicate this image and classify one of the duplicates as a promyelocyte and the other as a myelocyte. 2) If an image contains a clump of three Ebls, CellaVision often interprets it as a cell with a branched nucleus, for example, a segmented neutrophil. In this case, we must triplicate the image and classify it as Ebls.

**Supplementary Text 2 (Method)**

We are saving our data in a 2 terabytes external hard disk drive, and typically data obtained from 200 cases occupies 20-25 gigabytes.

**Supplementary Text 3 (Method)**

The study coordinators provided documents and videos that showed how to use the remote access software and CellaVision DM software and emphasised the following points.

1) This study does not compare data between participants, as evidenced by the overlap of patients between participants was minimal.

2) This study examines the consistency between paired data obtained by the digital and conventional microscopic methods in each participant.

3) Since the myeloblast percentage is a key data point, the definition of items not included in the cell count (such as smudge cells and artefacts) and myeloblasts was as constant as possible in performing the BM cell classification by the two methods.

4) Therefore, finish the allocated work within a minimum period, hopefully in a few weeks.

5) Although participants may take a long time in front of a large PC screen to classify cells using a digital method, it is recommended to take a similar amount of time per cell as in conventional microscopy.

**Supplementary Text 4**

**Background information and discussion about discordant diagnoses**

The percentages of BM blasts determined by cytomorphology is important in the current clinical practice but have unsolid side. In a study, in which five top MDS cytomorphologists classified the same 264 cells, which had been captured digitally from a BM smear, the percentages of blasts ranged from 58% to 68% [1]. Therefore, the data variation exists even if the same 264 cells are classified by experts. Moreover, in determining percentages of blasts in the BM, other concerns exist including the selection of areas of BM slides for cell classification and the size of denominator. In BM smears, partially damaged cells, cells with little cytoplasm, and cells look like apoptotic are not rare. These cells are a potential source to variate the denominator size among examiners (i.e., which cells should be included in or excluded from the denominator), and, therefore, may influence percentages of blasts. Furthermore, intraobserver variation exists even in the blast counts [2-3]. Moreover, if these difficulties are perfectly overcome, application of mathematical models indicate that blast counts near the cut-off points (5%, 10%, and 20%) will result in uncertainty of diagnosis and should be interpretated carefully [4].

When the above findings are taken into account, the difference in blast percentages observed between two methods in the present study may be acceptable variation. Nevertheless, data concordance between digital and conventional methods should be thoroughly examined in each institution before clinical use.

**Supplementary Text 5**

**Analysis and discussion about four samples (marked as “a-d” in TABLE 3) which resulted in discordant diagnoses**

We cannot analyze the cell identification in conventional light microscope retrospectively (which one was classified as what cell) and true data (the gold standard) of each BM sample is difficult to define. Therefore, it is difficult to verify why data from some BM samples were discrepant between the two methods. To take a clue, we compared the data determined by the digital and conventional methods for four samples, which showed discrepant results in blast percentages with clinical significance (marked as a-d in TABLE 3). In the sample “a”, main difference besides blast percentages was that lymphocyte percentages were more in the digital data compared with the microscopy data. In the samples “c and d”, main difference besides blast percentages was that erythroblast (Ebl) percentages were more in the microscopy data compared with the digital data. In the sample “b”, main difference besides blast percentages was that lymphocyte percentages and basophil percentages were more in the digital data compared with the microscopy data.

A fraction of lymphocytes and Ebls sometimes show morphological similarity to blasts. Moreover, partially damaged cells and cells with scanty cytoplasm, which are particularly not uncommon in lymphocytes, Ebls, and basophils, may be classified as smudges or cells should be counted (e.g., as apoptotic Ebls) depending on examiners. These difficulties may explain, at least in part, the discrepant results in these four cases.

When morphologists generate significantly discordant cell classification data between the two methods, it may be worth if they compare/examine their discordant data. They may be able to identify the cause of the discordance (e.g., apoptotic Ebls are excluded from the cell count) and create more accurate cell classification data.

**Supplementary Text 6**

**Discussion about advantage to soften labours and facilitating broader adoption of cytomorphology**

The conventional manual differential using an optical microscope does not take long time for people who are experienced and confident about this work. Meanwhile, for people who are responsible for many BM samples, which are first examined by other people who are not-fully experienced/trained, and for those people under training themselves, the present digital method softens their labours as follows. The above responsible people can revise the results and coach people in training in anytime using a PC monitor, meanwhile people in training are relieved from too much responsibility accompanied with their microscopic work and can ask questions to their instructors when they both have time. Moreover, the data in Supplementary Table 3 suggested that people in training can readily use the present method.

Cellavision takes approximately 40 minutes to scan four slides from each case; this potentially constitutes a problem for laboratories that receive many BM samples daily. Therefore, developing new equipment, such as one that has multiple sensors (cell captures) and more high-speed sensors, would be beneficial.

**Supplementary Text 7**

**Discussion about flow cytometry (FCM) as an example of sophisticated diagnostic technologies**

The percentages of myeloblasts determined by FCM (often called myeloid progenitors [%] to discriminate from myeloblasts in cytology) have been analysed using diverse protocols even by people who have much experience in MDS FCM [5,6]. In the paper by Kern et al. [5], among 327 patients with excess blasts (myeloblasts 5% or more by cytomorphology), myeloid progenitors by FCM were 3% or less in 116 patients (35%). Moreover, CD34 is a good marker for measuring myeloid progenitors in MDS, but not in de novo acute myeloid leukaemia [7]. Therefore, at this moment, blast quantification by cytomorphology is essential and FCM may help, but cannot replace, the cytomorphology [8].

**Supplementary Text 8**

**Discussion about morphology in the present digital analysis**

In analysing cell morphology, several differences in conditions exist between the optical microscope and our digital method. 1) The standard BM smears are prepared at the bedside using uncoagulated BM cells immediately after the aspiration, while the CV BM smears are prepared at our laboratory using EDTA-coagulated BM cells with or without dilution with plasma within one hour after the aspiration. 2) The CV BM smears are more spread than standard BM smears. It is probable that the mechanical forces on cells during smear preparation differ between the two kinds of smears. 3) During examining BM cells, the light intensity and contrast can be changed for optimizing to each cell and examiner in the optical microscope but not in the present digital method. 4) The CellaVision cannot capture enough number of megakaryocytes for assessing dysplasia.

Therefore, judging dysplasia using the current knowledges, which have been obtained through the light microscope data/experience, is not justified to apply for our digital data at this moment. Some features of dysplasia can be analysed in our digital data, provided that these features can be objectively compared between MDS samples and various non-MDS samples. Further studies are needed to achieve this goal.

The morphological differences shown in Figure 2C was probably induced by the above-mentioned differences in conditions between the optical microscope and our digital method. Although the judging the morphological difference is intrinsically subjective, we observed two cases (both are CD41-positive MDS), in which blast morphology differed as is Figure 2C, among 100 cases analysed in Table 2. This may be partly due to the fact that the blast morphology is often distinct in CD41+ MDS [9], and therefore, easy to identify their morphological change.

**REFERENCES (for Supplementary Texts)**

1. Mufti G et al. Diagnosis and classification of myelodysplastic syndrome: International Working Group on Morphology of myelodysplastic syndrome (IWGM-MDS) consensus proposals for the definition and enumeration of myeloblasts and ring sideroblasts. Haemtologica 2008. 93, 1712-1717
2. Browman GP et al. The contribution of cytochemistry and immunophenotyping to the reproducibility of the FAB classification in acute leukemia. Blood 1986. 68, 900-905
3. Chudgar U et al. Intraobserver and interobserver reproducibility of the FAB classification in acute leukaemia. Indian J Pathol Microbiol 1992. 35, 229-236
4. Vollmer RT. Blast counts in bone marrow aspirate smears: analysis using the Poisson probability function, Bayes theorem, and information theory. Am J Clin Patho 2009. 131, 183-188
5. Kern W et al. Multicenter prospective evaluation of diagnostic potential of flow cytometric aberrancies in myelodysplastic syndromes by the ELN iMDS flow working group. Cytometry Part B: Clinical Cytometry 2023.104, 51-65
6. Johansson U et al. The flow cytometry myeloid progenitor count: A reproducible parameter for diagnosis and prognosis of myelodysplastic syndromes. Cytometry Part B: Clinical Cytometry 2023. 104, 115-127
7. Ogata K et al. Clinical significance of phenotypic features of blasts in patients with myelodysplastic syndrome. Blood 2002. 100, 3887-3896
8. Hasserjian RP et al. Diagnosis and classification of myelodysplastic syndromes. Blood 2023. 142, 2247–2257
9. Ogata K et al. Clinical, immunophenotypic, and cytogenetic characteristics of high-grade myelodysplastic syndromes with CD41-positive progenitor cells. Cytometry Part B: Clinical Cytometry. 2023. 104, 98-107
